# Challenges and advances in materials and fabrication technologies of small-diameter vascular grafts

**DOI:** 10.1186/s40824-023-00399-2

**Published:** 2023-06-08

**Authors:** Mei-Xian Li, Qian-Qi Wei, Hui-Lin Mo, Yu Ren, Wei Zhang, Huan-Jun Lu, Yoon Ki Joung

**Affiliations:** 1 National and Local Joint Engineering Research Center of Technical Fiber Composites for Safety and Protection, Nantong University, Nantong, 226019 China; 2 School of Textile and Clothing, Nantong University, Nantong, 226019 China; 3 Center for Biomaterials, Biomedical Research Institute, Korea Institute of Science and Technology, Seoul, 02792 Republic of Korea; 4 Department of Infectious Diseases, General Hospital of Tibet Military Command, Xizang, China; 5 Institute of Special Environmental Medicine, Nantong University, Nantong, 226019 China; 6 Division of Bio-Medical Science and Technology, University of Science and Technology (UST), 217 Gajeong-ro, Yuseong-gu, Daejeon, 34113 Republic of Korea

**Keywords:** Small-diameter vascular grafts, Surface modification, Cardiovascular diseases, Biomimetics, Tissue engineering

## Abstract

The arterial occlusive disease is one of the leading causes of cardiovascular diseases, often requiring revascularization. Lack of suitable small-diameter vascular grafts (SDVGs), infection, thrombosis, and intimal hyperplasia associated with synthetic vascular grafts lead to a low success rate of SDVGs (< 6 mm) transplantation in the clinical treatment of cardiovascular diseases. The development of fabrication technology along with vascular tissue engineering and regenerative medicine technology allows biological tissue-engineered vascular grafts to become living grafts, which can integrate, remodel, and repair the host vessels as well as respond to the surrounding mechanical and biochemical stimuli. Hence, they potentially alleviate the shortage of existing vascular grafts. This paper evaluates the current advanced fabrication technologies for SDVGs, including electrospinning, molding, 3D printing, decellularization, and so on. Various characteristics of synthetic polymers and surface modification methods are also introduced. In addition, it also provides interdisciplinary insights into the future of small-diameter prostheses and discusses vital factors and perspectives for developing such prostheses in clinical applications. We propose that the performance of SDVGs can be improved by integrating various technologies in the near future.

**Graphical Abstract:**

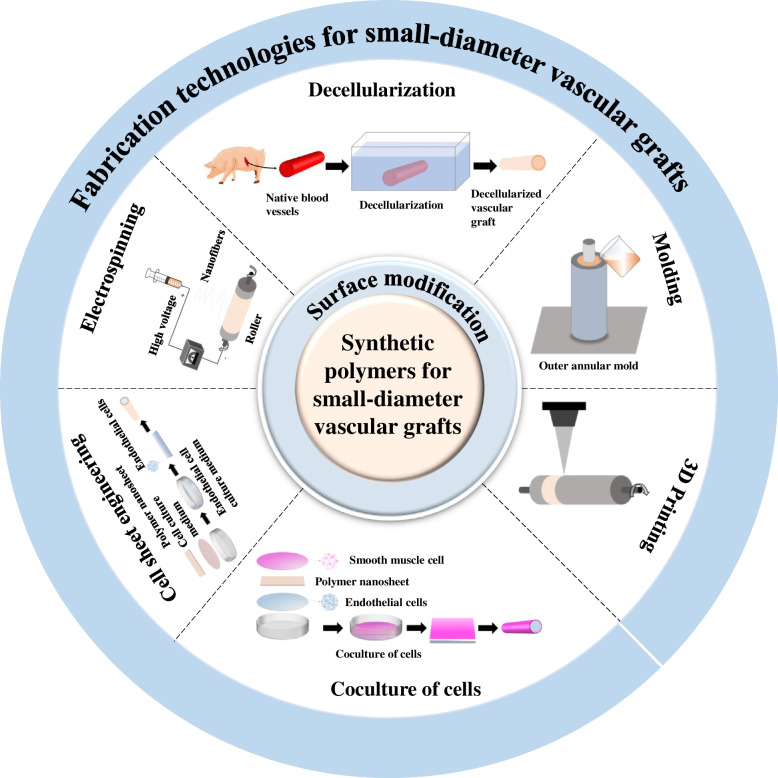

## Highlights

• The commonly used synthetic polymers for vascular grafts are reviewed.

• Various methods for surface modification of artificial vascular grafts are summarized.

• Latest fabrication techniques for small-diameter vascular grafts and their mechanical and biological properties are systematically summarized.

• Current state and future perspective of small-diameter vascular grafts are suggested.

## Introduction

**Fig. 1:**
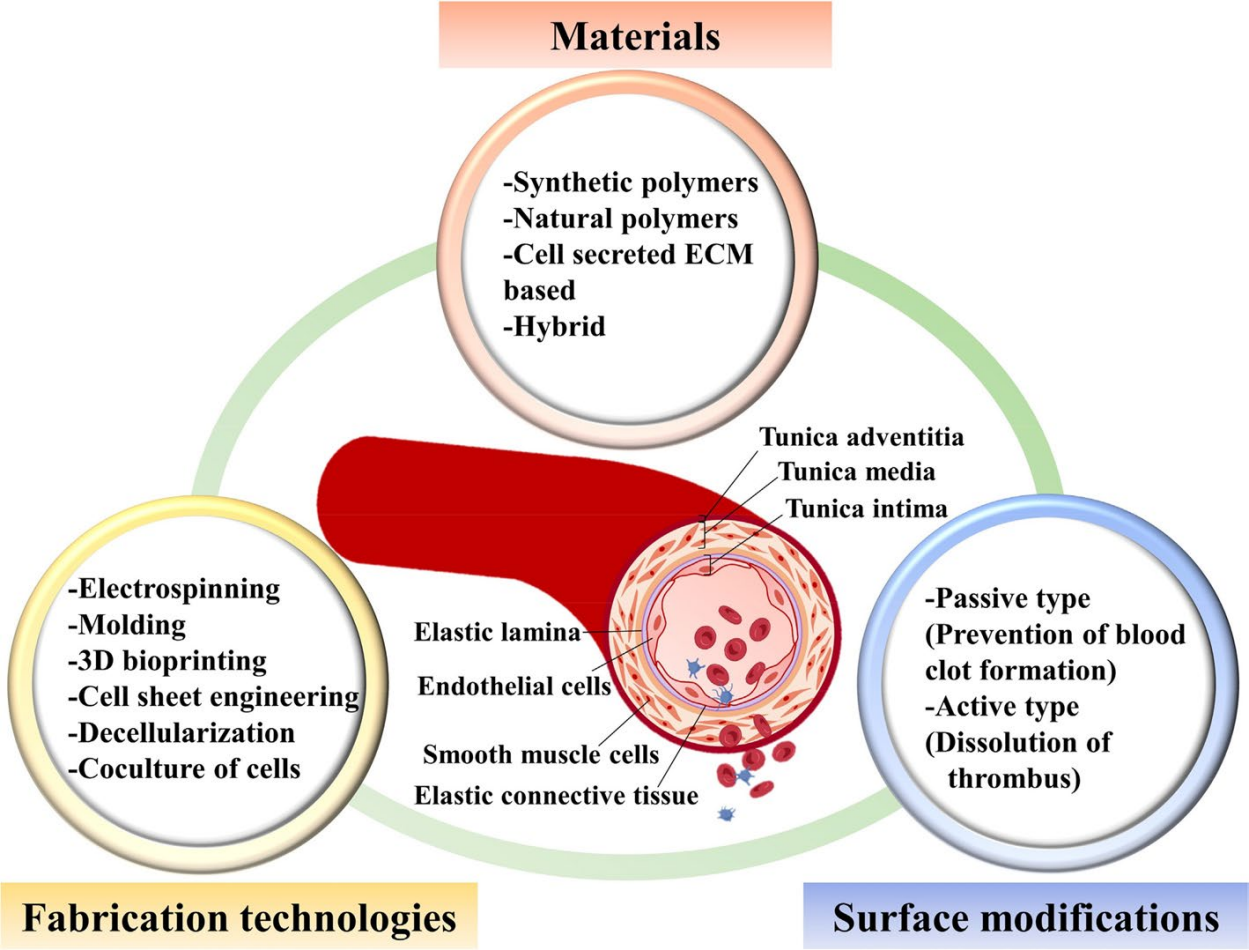
Brief outline of various considerations for SDVGs discussed in this review, including commonly used materials, surface modification strategies, and various fabrication technologies

Cardiovascular diseases have one of the highest morbidities and mortality rates in the world, with an estimated 12.1 million to 18.6 million deaths in the past 30 years [1]. The most common cardiovascular diseases include coronary artery diseases, deep vein thrombosis, and myocardial infarction, usually associated with stenosis and embolism of blood vessels [2–4]. Bypass of grafting using autologous blood vessels, allogenic blood vessels, or artificial blood vessels is one of the most preferred treatments for cardiovascular diseases. The most optimal treatment among them is autologous blood vessels due to their adequate size and good biocompatibility for long-term patency. However, insufficient supply and trauma of the donor site limit its clinical application. In addition, the grafts of allogenic blood vessels are associated with potent immune responses, leading to the rejection of a graft. Therefore, artificial blood vessels might potentially replace or bypass diseased blood vessels. It is convenient to prepare an artificial vascular graft with a suitable diameter and length. The ideal vascular grafts should meet the following requirements: biocompatibility, mechanical properties, permeability, and anti-thrombosis [5, 6]. A vascular graft should be histo-compatible to resist immune responses and rejection. It must also have satisfactory mechanical properties to retain its integrity and to withstand physiological pressures. Furthermore, the microstructure should be conducive to nutrient exchange, elimination of metabolic products, and cell growth, allowing endothelial cells to attach, proliferate, and form a endothelial layer with tight junction, capable of resisting thrombosis. Nowadays, artificial vascular grafts for larger size diameters (> 6 mm) are used in the global vascular grafts market. In contrast, there is still no successful clinical trial for SDVGs (< 6 mm) which are in great demand for the treatment of various arterial complications such as coronary artery disease and pediatric congenital cardiovascular defects. The main reason is the intimal hyperplasia at the anastomotic site caused by compliance mismatch between SDVGs and native vessels as well as thrombus formation on the synthetic surface of SDVGs, resulting in low patency after implantation [7–9]. To overcome these limitations, various innovative approaches, including ideas such as their structures, surface modifications, and mechanical and biological requirements, are being adopted to mimic native vessels for the long-term patency of SDVGs [10–12]. Native blood vessels have a complex structure with three distinct layers, the inner layer (tunica intima), the middle layer (tunica media), and the outer layer (tunica adventitia), which significantly affects the mechanical and biological properties (Fig. 1) [13]. The inner layer, known as the endothelium, is the thinnest in the structures of a blood vessel, which acts as a barrier controlling the passage of biologically active substances and selectively penetrates fluids, ions, molecules, and leukocytes. The middle layer is relatively thick, mainly determined by the number of smooth muscle layers supported by elastic connective tissues. The smooth muscles and elastic fibers regulate the contraction and dilation of blood vessels. Meanwhile, a framework of collagen fibers in the middle layer binds the layers to the inner and outer layers of blood vessels and maintains the tension of blood vessels. The outer layer is generally thinner than the middle layer, primarily consisting of connective tissues with elastic and collagen fibers. It plays a vital role in supplying the vessel wall with nerves and self-vessels and helping to hold the vessels to surrounding tissues. Overall, to mimic native blood vessels, vascular graft constructs must be able to efficiently and selectively exchange nutrients and waste with all cells and meet mechanical requirements.

Furthermore, bioactive substances are introduced into the constructs for excellent biocompatibility and inducible endothelialization. Generally, the synthetic polymers that have been used as artificial vascular grafts lack bioactivity, and it is challenging to realize rapid endothelialization due to the low initial adhesion of endothelial cells. Therefore, the improvement of bioactivity in vascular grafts during fabrication is one of the most challenging subjects. Current approaches to this include the local delivery of angiogenic growth factors (vascular endothelial growth factor (VEGF), fibroblast growth factor (FGF), hepatocyte growth factor (HGF)) [14–16], the transplantation of stem and progenitor cells as well as angiogenic genes [17–19], and so on. Despite the success of these strategies in promoting endothelial cell adhesion to reduce thrombosis and intimal hyperplasia, there are still some drawbacks.

This review appraises a comprehensive overview of the materials with surface modification and fabrication technologies for SDVGs. First, we briefly introduce the synthetic polymers used for vascular grafts and their surface modification combined with their structures. We further describe the latest techniques and strategies for the fabrication of SDVGs (Fig. 1). Finally, the current status of the related researches and future perspectives will be addressed.

## Materials and their surface modification for SDVGs

In general, both natural polymers and synthetic polymers are used for vascular grafts. Natural polymers, such as collagen, elastin, gelatin, and lecithin, comprised of the components of the extracellular matrix (ECM), provide many necessary signals for cell functions. They, therefore, have adequate biocompatibility and are likely to be less rejected by the immune system. However, their low mechanical properties and rapid degradation limit their individual use for vascular grafts. Therefore, these natural polymers are usually mixed with synthetic polymers to mimic native blood vessels that, give rise to better cell attachment and proliferation contributed by natural polymers, and meet mechanical requirements for native blood vessels owing to good mechanical properties of synthetic polymers [20–22]. It is reported that blending proper ratios of collagen, elastin, and synthetic polymers improves tensile strength and Young’s modulus and exhibits a good biocompatibility [23]. Herein, we introduce several commonly used synthetic polymers as materials for vascular grafts.

### Commonly used synthetic polymers for vascular grafts

Considering the commercialization of large-diameter synthetic vascular grafts with more than 90% patency after one-year implantation [24], researchers have focused on available synthetic polymers used for SDVGs. The most commonly used synthetic polymers for vascular grafts are expanded polytetrafluoroethylene (ePTFE), polyethylene terephthalate (PET), polyurethane (PU), polylactic acid (PLA), poly(-caprolactone) (PCL), poly (glycolic acid) (PGA), and some hybrid polymers. An overview of these synthetic polymers and their properties for vascular grafts are compared with rat aorta and listed in Table 1 [25–33].

**Table 1:** Overview of graft materials

Graft materials	Molecular weight	Polymer solution concentration	Inner Diameter (mm)	Burst pressure (mmHg)	Ref
Rat aorta	-	-	-	1043 ± 326	[30]
Human arteries	-	-	-	2031 ~ 4225	[27]
ePTFE	-	-	1.0	2715 ± 33	[28]
PET (Dacron®)	-	-	-	4145 ± 207	[26]
Nonwoven PET	-	-	4.7	1509 ± 175	
PU	-	15 wt% in THF/DMF (3:1 v/v)	2.0	1156 ± 149	[29]
PU/PCL	1:1 ratio	10 wt% in chloroform/ethanol (7:3); 15 wt% in THF/DMF (3:1 v/v)	2.0	2017 ± 72	
PCL	80 kDa	15% in CHCl_3_/EtOH (70% v/v)	2.0	3280 ± 280	[32]
PLLA/PCL	130 kDa	6% in 2, 2, 2-trifluoroethanol	1.0	3175 ± 438	[25]
PET/PU	4:1 ratio	18 wt% in HFIP	4.0	1787.64 ± 76.61	[33]

Chloroform (CHCl_3_); ethanol (EtOH); N,N-dimethylformamide (DMF); tetrahydrofuran (THF); 1,1,1,3,3,3-hexafluoro-2-propanol (HFIP)

#### Expanded polytetrafluoroethylene (ePTFE)

**Fig. 2:**
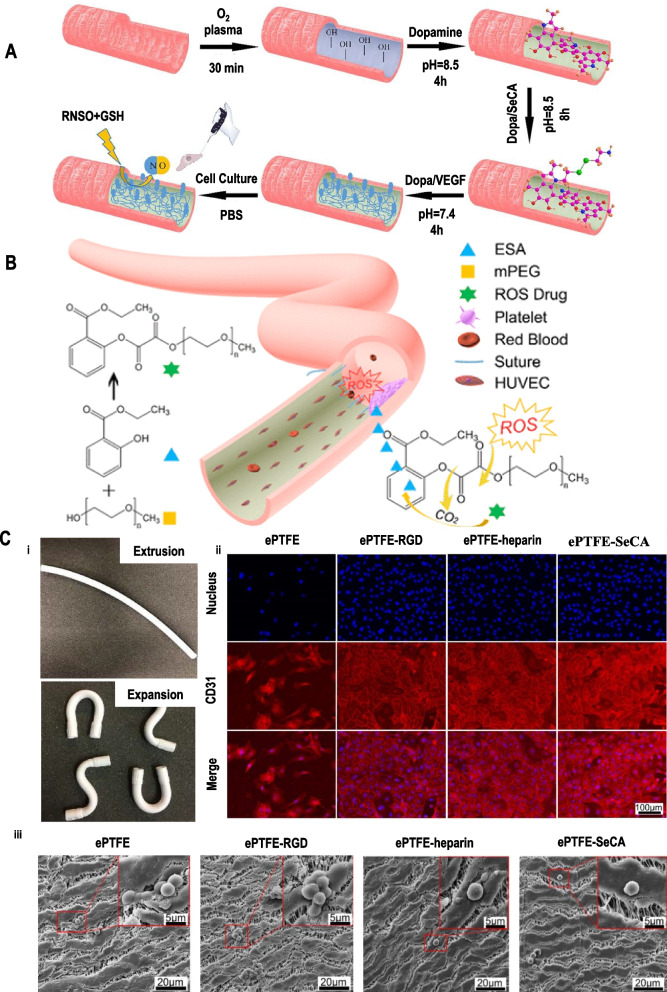
(**A**) A schematic of the ePTFE surface modification process to obtain ePTFE-dopamine/ selenocystamine (SeCA)/VEGF (ePTFE-DSV). Reproduced with permission [41]. Copyright 2020, Elsevier. (**B**) Synthesis route of the reactive oxygen species-responsive drug. Reproduced with permission [40]. Copyright 2020, American Chemical Society. (**C**) i) Tube product of the extrusion process and expansion process. ii) Platelet adhesion of ePTFE grafts, ePTFE-RGD, ePTFE-heparin, ePTFE-SeCA. The scale bar is 100 μm. iii) Immunochemical staining of HCAECs cultured on ePTFE, ePTFE-RGD, ePTFE-heparin, and ePTFE-SeCA grafts at day 10. The images show the nuclei (blue) and the expression of CD31 (red). The scale bar is 20 μm. Reproduced with permission [39]. Copyright 2020, American Chemical Society

Expanded polytetrafluoroethylene (ePTFE) is obtained by structurally modifying PTFE to maintain its good chemical stability. The ePTFE-based large-diameter vascular grafts emerged in 1974 with desirable mechanical strength, excellent resistance to degradation, and satisfactory patency (Fig. 2C (i)), leading to ePTFE (Gore-Tex®) being commercially used as large diameter (> 6 mm) vascular grafts [24]. However, it prevents endothelial cell adhesion and leads to platelet activation due to the hydrophobic surface, resulting in thrombosis, poor patency, and low compliance for SDVGs [34]. Therefore, recent studies focus on modifying the surface of ePTFE to prohibit thrombosis, restenosis, and rapid endothelialization (Fig. 2A, B). For example, ePTFE surfaces are modified by functional biomolecules, plasma treatment, the immobilization of proteins (collagen and elastin), or binding of anticoagulants (heparin), and so on [35–41]. These modifications successfully promote endothelialization and prevent platelet adhesion and thrombosis, providing the basis for the further clinical application of SDVGs (Fig. 2C (ii-iii)).

#### Polyethylene terephthalate (PET)

Polyethylene terephthalate (PET, Dacron®), with high elastic modulus, mechanical stability, and excellent tensile strength, is one of commercially used synthetic polymers for large-diameter vascular grafts. Generally, PET is divided into woven fibers (Dacron®) and nonwoven PET, of which the former is less porous than the latter, directly affecting mechanical and biological properties (Table 1). The Dacron has been used for large diameter vascular grafts for 60 years as it has the higher patency than 85% at five years [42, 43]. Although it has excellent mechanical properties and high patency for large-diameter vascular grafts, some limitations exist if it is used for tiny-diameter vascular grafts. For example, the hydrophobic surface of PET, like that of ePTFE, resists endothelial cell adhesion and cell proliferation, causing platelet activation and thrombosis [44]. Therefore, similar approaches to the surface modification of ePTFE (such as plasma treatment, binding anticoagulants, or bioactive proteins) have been taken for the PET-based vascular grafts to decrease their surface hydrophobicity and enhance the cell adhesion and growth, achieving antithrombosis and rapid endothelialization.

#### Polyurethane (PU)

Polyurethane (PU) has a linear block structure composed of a soft segment (polyether or polyester polyol) and a hard segment (chain extender and diisocyanate) that alternately connects to the main chain with carbamate groups. The microphase separation structure of PU is obtained by the difference in the polarity of soft segments (low polarity) and hard segments (high polarity), facilitating superior biocompatibility, compliance, elasticity, and anticoagulation of PU compared with ePTFE and Dacron. The mechanical properties of the PU-based vascular grafts are comparable with native blood vessels, and the endothelialization rate of PU is faster than that of ePTFE and Dacron. In addition, the layer composed of new cells is thinner, facilitating better patency. However, its clinical application is limited by poor biostability and spontaneous degradation at the early implantation stage. Researchers have developed new generation of PU grafts using soft polycarbonate segments to improve their biostability in vivo, showing no degradation of polycarbonate PU during the early six months and faster endothelialization compared with ePTFE grafts. Despite improvements in mechanical and biological properties of PU through surface modifications (grafting functional groups or binding anticoagulants) and combined the structures with bioactive factors, the patency rate was less than 60% after 12 months, which is not enough to satisfy the clinical requirements [45]. Therefore, more advanced methods must be explored to form more reliable methods for SDVGs.

#### Poly(ɛ-caprolactone) (PCL)

Poly(ɛ-caprolactone) (PCL) consists of repeating units with one polar ester group and five non-polar methylene groups, and has excellent biodegradability and biocompatibility. The ester group in the PCL is broken down into water and carbon dioxide, forming less acid decomposition products, and it exhibits relatively slow degradation (more than two years) compared to other synthetic biodegradable polymers, providing sufficient time for cell adhesion, cellular ingrowth, and tissue regeneration [46]. However, the long-term patency for SDVG is still a challenge. Studies on modifying PCL vascular grafts to improve their mechanical properties, biocompatibility, and patency has been in progress. Chen et al*.* [47] showed that bioinspired zwitterionic poly[2-(methacryloyloxy)ethyl choline phosphate] treated PCL significantly reduced nonspecific protein adsorption and remarkably improved the adhesion and proliferation of the cells. Cuenca et al*.* [48] fabricated PCL SDVGs comprised of decellularized extracellular matrix with high mechanical properties, showing less hemolysis and low blood coagulation behavior than non-treated PCL. Though PCL vascular grafts exhibited complete endothelialization by 12 weeks and retained good patency without aneurysmal dilation or thrombosis until 18 months in the abdominal aorta of a rat model, a regression in cellular infiltration is observed, followed by calcification lesions after 18 months [32]. Therefore, it needs further investigations to be established as a long-term patent PCL vascular graft.

#### Polylactic acid (PLA)

Polylactic acid (PLA) is an aliphatic polyester derived from renewable resources with two optical isomers, poly-D-lactic acid (PDLA) and poly-L-lactic acid (PLLA), where PLLA is more commonly used in the medical materials [49]. It is reported that PLA deposition product is non-toxic and easily degraded into carbon dioxide and water without triggering an immune response in the body. Hence, it is considered an ideal material for biodegradable vascular grafts. Khalifehzadeh et al*.* [50] modified the surface of PLA with a perfluoro compound facilitated by surface activation by radio frequency plasma to reduce thrombogenicity and platelet reactivity. However, the cell affinity of PLA is relatively poor, making it challenging to adhere, proliferate and differentiate endothelial cells onto the surface of the vascular grafts. However, the weak mechanical properties of PLA and the rapid degradation rate (two months) limit its vascular application. This led to the researchers developing PLA composites compounded with other polymers, including natural or synthetic polymers, to enhance their mechanical properties and control their degradation rate [51, 52]. In addition, complete endothelialization before degradation requires improved in vitro bioactivity before implantation.

### Types of surface modifications

**Fig. 3:**
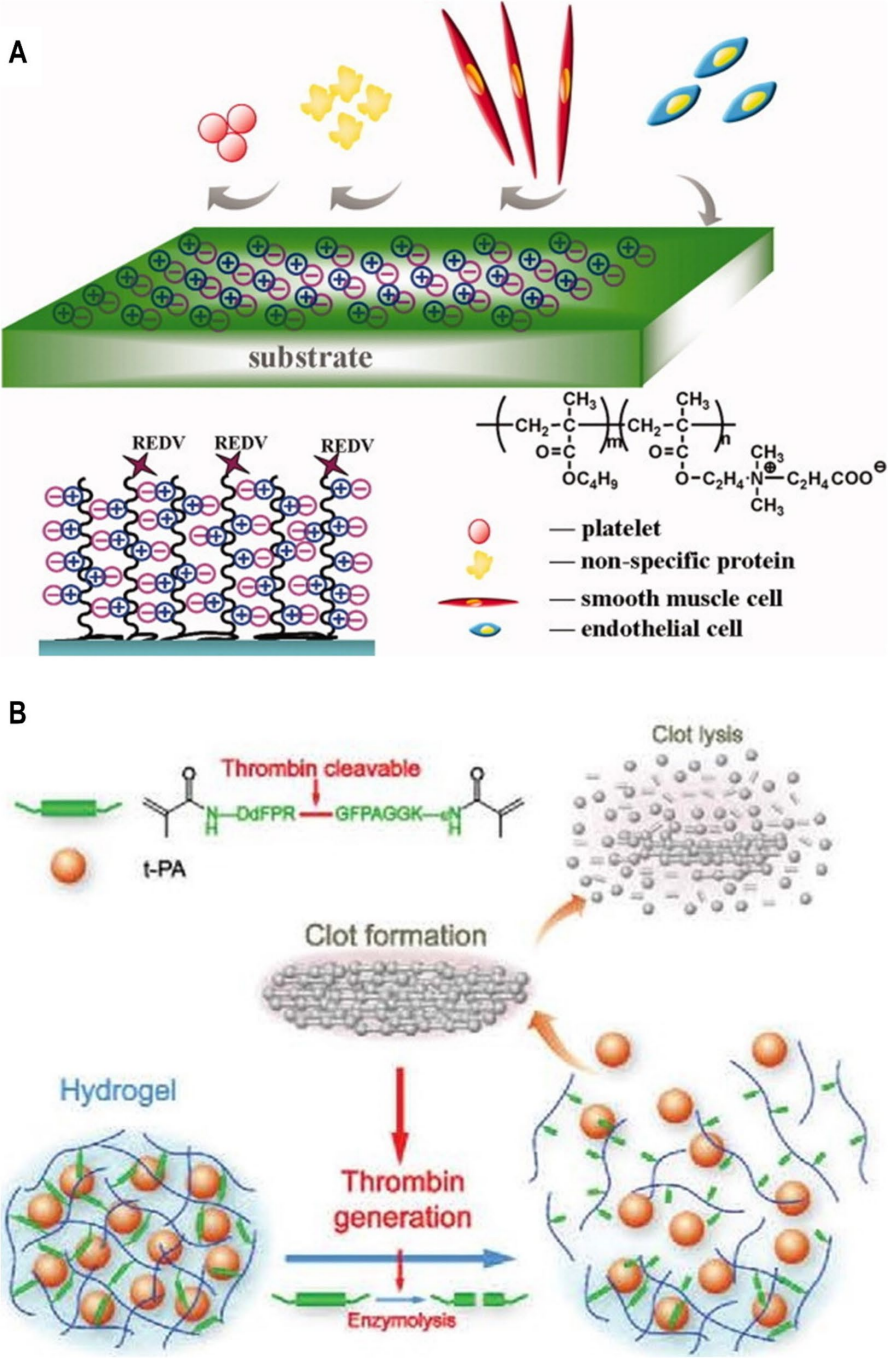
Illustration of the mechanism of (**A**) passive type modification (carboxy betaine-REDV polymer coating). Reproduced with permission [54]. Copyright 2012, John Wiley and Sons. (**B**) active type modification (thrombin-responsive clot-lysing activity). Reproduced with permission [55]. Copyright 2014, Royal Society of Chemistry

Native blood vessels of the human body are composed of three layers mentioned before, each has different functions. Therefore, only using the original synthetic polymer materials could not satisfy the parts of blood vessels. Studies on modifications to the luminal surface and the introduction of biologically active substances for SDVGs are in the spotlight to mimic the multiple performance requirements for native blood vessels. Buscemi et al*.* [51] modified polycaprolactone (PCL) with alpha, beta-poly(N-2-hydroxyethyl)-D, and L-aspartamide, which blended with PLA and linked with heparin, showing excellent biocompatibility and moderate elastic tension to blood pressure in a pig model. Nguyen et al*.* [53] fabricated multilayer artificial blood vessels with PCL-gelatin as the inner layer, poly(lactic-co-glycolic acid)(PLGA)-gelatin as the middle layer, and PLGA-chitosan as an outer layer, which displayed excellent flexibility, cell attachment, and cell proliferation. The surface modification strategies expedited in situ endothelialization, eventually reducing thrombosis and intimal hyperplasia. Overall, surface modification of synthetic polymer vascular grafts can be divided into passive type modification (prevention of blood clot formation) and active type modification (dissolution of thrombus) according to the mechanism of antithrombosis (Fig. 3).

#### Passive type modification

Late endothelialization is a critical problem of SDVGs caused by the low initial adhesion of endothelial cells and the difficulty of in situ endothelialization. This problem needs immediate attention to be solved. To enhance endothelial cell adhesion and growth environment, many EC-adhesive ligands, including peptides (RGD, CAG, REDV, and YIGSR), vascular growth factors (VEGFs, FGFs, and HGFs), and antibodies, have been used in artificial vascular grafts. They have also been used in artificial vascular scaffolds, promoting endothelial cell adhesion and proliferation, thus reducing thrombosis and intimal hyperplasia, and improving its patency rate, providing an adhesion and growth environment for endothelial cells (Fig. 3A) [54, 56]. Ding et al*.* [57] modified a polyurethane surface with REDV peptide to enhance the endothelialization of implants. The process needs three days to form a layer of primary human umbilical vein endothelial cells (HUVECs) on the PU/REDV surfaces. In contrast, the proliferation of primary human umbilical artery smooth muscle cells (HUASMCs) was inhibited. Moreover, the PU/REDV surface reduced the activity of adhered platelets, selectively regulated HUVECs, and delayed the proliferation of human umbilical artery ECs (HUAECs).

Tissue-engineered vascular grafts would induce the body to recognize them as autologous, decreasing the risk of rejection [58]. This modification thus improves the anti-thrombosis of vascular grafts; however, it still needs further development for the long-term patency of SDVGs.

#### Active type modification

Extensive studies have been performed to elucidate different methods of surface modification and their role in antithrombosis. Yet limited attempts have been made to modulate thrombosis in the initial implantation stage. The lack of local tissue fibrinolytic activity, capable of dissolving intravascular thrombosis during the early stage of implantation, might be one of the attributes responsible for the failure of vascular graft implantation [59]. Subsequently, many groups have attempted to develop thrombin-sensing materials. Gunawan et al*.* [60] designed a thrombin-sensing fabric based on thrombin formation during blood coagulation. They developed bright poly(2-oxazoline) based multifunctional polymer that was degraded by serine protein thrombin and released the urokinase plasminogen activator to target the focal area of thrombosis to dissolve the thrombus selectively. Du et al*.* [55] also developed a thrombin-triggering thrombolytic material, which act when a clot or thrombus formation and effectively prevented thrombosis formed at the early stage (Fig. 3B). Lin’s group [61] developed a bio-responsive peptide-polysaccharide nano-complex which could self-titrate the release of anticoagulants depending on varying levels of coagulation activity through negative feedback regulator. The nano-complex circulates are active only under thrombotic conditions with releasing anticoagulant. The nano-complex locally releases its drug payload and prevents thrombosis in response to life-threatening pulmonary embolism.

Although the surface modification techniques of vascular graft materials have become highly advanced and have already been commercialized in the large diameter, their long-term patency effect in SDVGs is still not ideal. The problems of thrombosis and intimal hyperplasia have not been fully resolved, failing to establish a causative relationship.

## Fabrication technologies

The problems of cell adhesion, proliferation, and nutrient transport warrant tissue-engineered vascular grafts to be porous, such as fiber diameter and pore size. There are many methods to fabricate porous vascular grafts, such as electrospinning [62–65], phase separation [66, 67], freeze-drying [68, 69], 3D printing [70, 71], and so on. The advantages and disadvantages of each fabrication technology have been listed in Table 2.

**Table 2:** The advantages and disadvantages of various fabrication methods used for SDVGs

Scaffold Fabrication Method	Advantage	Disadvantage	Ref
Electrospinning	– Mimic similar fibrous architecture of native extracellular matrix structure with high porosity to provide excellent biocompatibility including cell adhesion and proliferation – Increase mechanical properties – Fabricate multilayer grafts	– Use toxic solvent and high voltage – Unable to fabricate complex structures – Coaxial electrospinning cause interface effects	[72–77]
Molding	– Easy to setup – Fabricate multilayer grafts – Fabricate various geometries and dimensions with micropatterned surfaces – Control porosity and pore size by tuning the processing parameters	– Use toxic solvent – Need for mechanical reinforcement	[78–82]
3D bioprinting	– Automatic process with good controllability, reproducibility, and repeatability, including pore structures of vascular grafts – Incorporation of cells spatially	– Limited materials – Slow to fabricate mass customization – Need for post-processing – Unable to fully mimic the complex structure and function of natural vasculature	[83–85]
Cell sheet engineering	– Reduce immune response after implantation by using autologous cells – Biologically mimic native extracellular matrix	– Unable to use readily due to production time – Potential for delamination failure	[86–88]
Decellularization	– Preservation of the extracellular matrix – Favorable mechanical properties	– Unable to use readily due to production time – Potential for immune responses – Difficulty in precise recellularization – High cost – Low cellularity upon implantation – Donor tissue loss if an autologous graft is used	[89–92]
Coculture of cells	– Mimic the cellular environment in the body including cell–cell interactions and cell-culture interactions – Induce cell differentiation to improve the speed of vascularization	– Difficulty in precise control over cell–cell interactions and separation for implantation	[93–95]

### Electrospinning

**Fig. 4:**
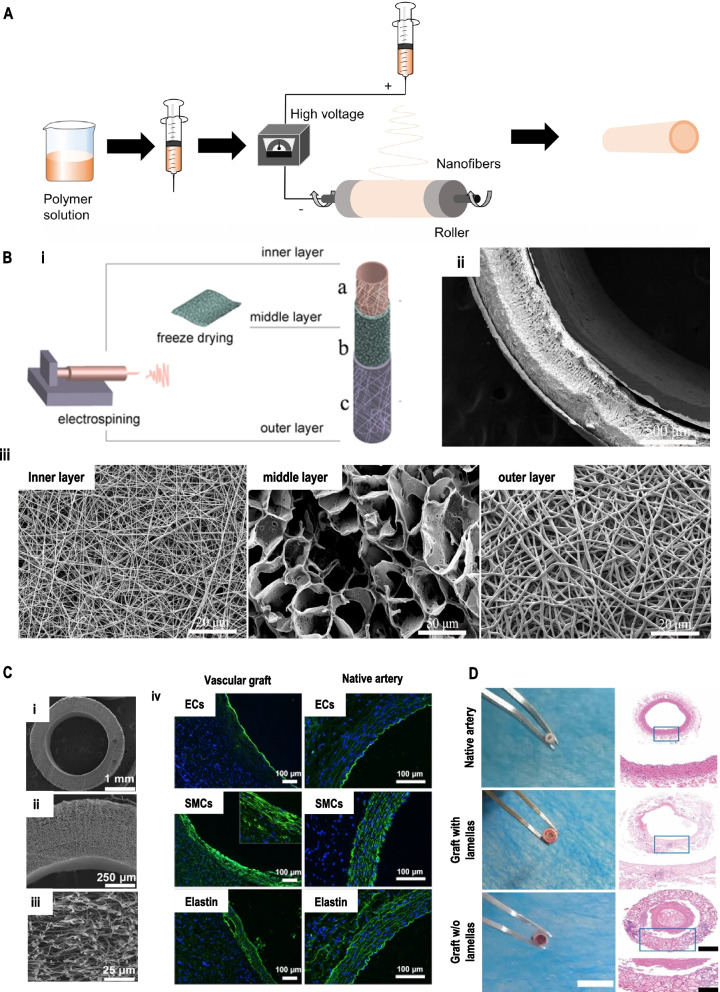
(**A**) Fundamental schematic diagrams of electrospinning. (**B**) Schematic illustration of i) the construction process of the tri-layer scaffold and, ii) SEM images of the cross-section of the tri-layer scaffold, iii) high magnification image of the inner layer, middle layer, and outer layer, respectively. Reproduced with permission [72]. Copyright 2020, Elsevier. (**C**) i-iii) SEM images of electrospun PCL mats with thinner-fiber grafts, iv) cross-sectional images of the regenerated and native artery were immuno-stained to detect the endothelial cells, smooth muscle cells, and elastin. Reproduced with permission [97]. Copyright 2018, Jove (**D**) Optical images and hematoxylin–eosin (H&E) staining images of a cross-section of native vessel and graft after three months. Reproduced with permission [98]. Copyright 2019, American Chemical Society

In the field of textile science, the technologies for the fabrication of vascular grafts include not only electrospinning but also knitting and weaving methods. Knitted vascular grafts have greater porosity than woven, encouraging infiltration of cells and capillary formation. This leads to better matching of the knitted vessels with the native ones and reduced intimal hyperplasia. However, large pore size, the swelling phenomenon of knitted vascular grafts, and the surgical difficulty of woven vascular grafts make it challenging to fabricate SDVGs. Among the available technologies, electrospinning is considered ideal for fabricating vascular grafts because the fiber fineness, thickness, three-dimensional structure, mechanical properties, and even degradation rate can be controlled by the adjustment of the parameters (Fig. 4A). Moreover, various types of polymers can be used for electrospinning. Different cross-sections of fibers (e.g., co-axial or tri-axial structure) are prepared by multiple needles and polymer solutions (Fig. 4B) [72, 96]. In the fiber morphology, continuous ultrafine fibers with diameters from 10 to 100 nm are fabricated by an electrospinning process, with large specific surface areas to benefit the mimicry of natural extracellular matrix (ECM) for cell adhesion, proliferation, and differentiation exhibiting excellent cell-compatibility. The luminal surface of appropriate vascular grafts is covered by newly formed endothelium similar to that of the native vessel with the regeneration of media and outer layer (Fig. 4C). In addition, bioactive factors are introduced to the vascular grafts and evenly distributed on the surface of the fibers, leading to widespread use in bone, nerve, and vascular graft repairs.

In general, the morphology and the properties of fibers are affected by various factors, such as molecular weight and cross-linking degree of polymers, the viscosity of the solution, solvent volatility, applied static voltage, receiving distance, temperature, humidity, and so on. Xiang et al*.* [99] used the electrospinning technique to fabricate a tubular scaffold composed of recombinant silk protein/PCL/gelatin with a 3 mm inner diameter, which showed high porosity leading to good cell growth and proliferation. Aydogdu et al*.* [100] prepared a novel biomimetic PCL/ethyl cellulose/collagen type-1 based SDVGs scaffold using the electrospinning method, which creates the most convenient synergy of a natural and synthetic polymer. This leads to the achievement of similarity to native small-diameter blood vessels with proper Young’s modulus and cell viability. In addition, Wang et al*.* [101] proposed a vascular graft by modifying its geometric structure. A spiral groove structure on a vascular graft was designed, a circular arc extending continuously on the inner wall. The transformation of the geometric system changed the blood flow distribution and increased the blood flow velocity near the border. Constant blood flow conditions lead to reduced deposition of harmful substances in the wall of the vascular grafts by creating a scouring force on the inner wall of the blood vessel. Moreover, increasing the shear force on the wall of the blood vessel leads to a long-term patency rate of the artificial blood vessel, especially the SDVGs (Fig. 4D). Furthermore, vascular grafts could be fabricated by combining one or two technologies; for example, fabrications can be achieved by a combination of electrospinning with the freeze-drying method, phase separation, molding method, or tissue engineering techniques [58, 72, 98, 102, 103]

### Molding

**Fig. 5:**
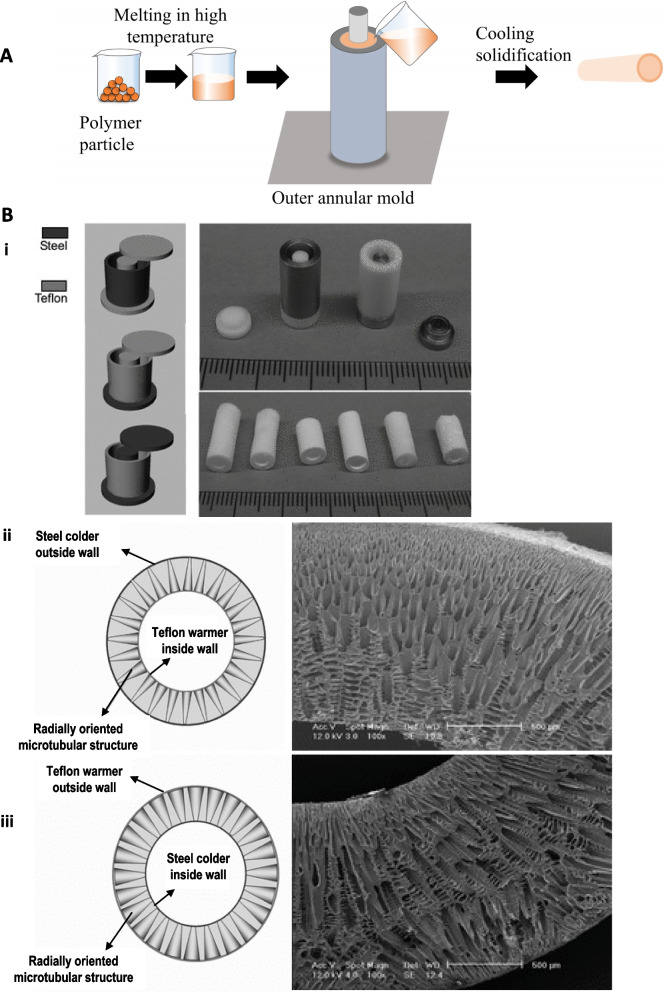
(**A**) Fundamental schematic diagrams of molding. (**B**) i) View of the molds used for preparing the oriented gradient microtubule-structured scaffolds: (left) schematic illustrations of the molds; (top right) appearance of the molds with two different materials; (bottom right) appearance of produced scaffolds. Schematic diagram and SEM images of cross sections of vessel scaffolds prepared under a radial temperature gradient at -20 ℃, ii) I/O structure, iii) O/I structure from PLLA (10%)/benzene solutions. Reproduced with permission [78]. Copyright 2010, John Wiley and Sons

Molding is also a traditional technology that generates tissue-engineered vascular grafts (Fig. 5A). It requires an easy set-up and allows the fabrication of customized shape (tubular-shape vascular grafts or complex branch vasculatures) scaffolds using polymers (Table 2) [104]. The polymer solution is poured into a designed mold with a fixed size of inner diameter and wall thickness of the vascular grafts (Fig. 5). A combination of technologies is required to generate suitable pores, such as gas foaming, phase separation, and among these methods, a thermal-induced phase separation is a common approach. Ma’s group [78] developed a SDVG using PLLA by the combination of molding and a thermally induced phase separation method, which allowed controlling the porosity and tubular size and was conducive to cell seeding and mass transfer for cell growth and function (Fig. 5B).

Moreover, it is demonstrated that varying processing parameters, such as the polymer concentration and temperature gradient, can control oriented microtubular structure. Zhen et al*.* [105] developed precision-engineered porous vascular grafts with crosslinked polyurethane which have excellent mechanical properties compared with native blood vessels. The results showed how pore size could significantly affect angiogenesis and cellularization, and polyurethanes with 40 μm pores have the highest level of angiogenesis and cellularization than that with 100 μm pores and non-porous structure, indicating the importance of pore size.

### 3D bioprinting

**Fig. 6:**
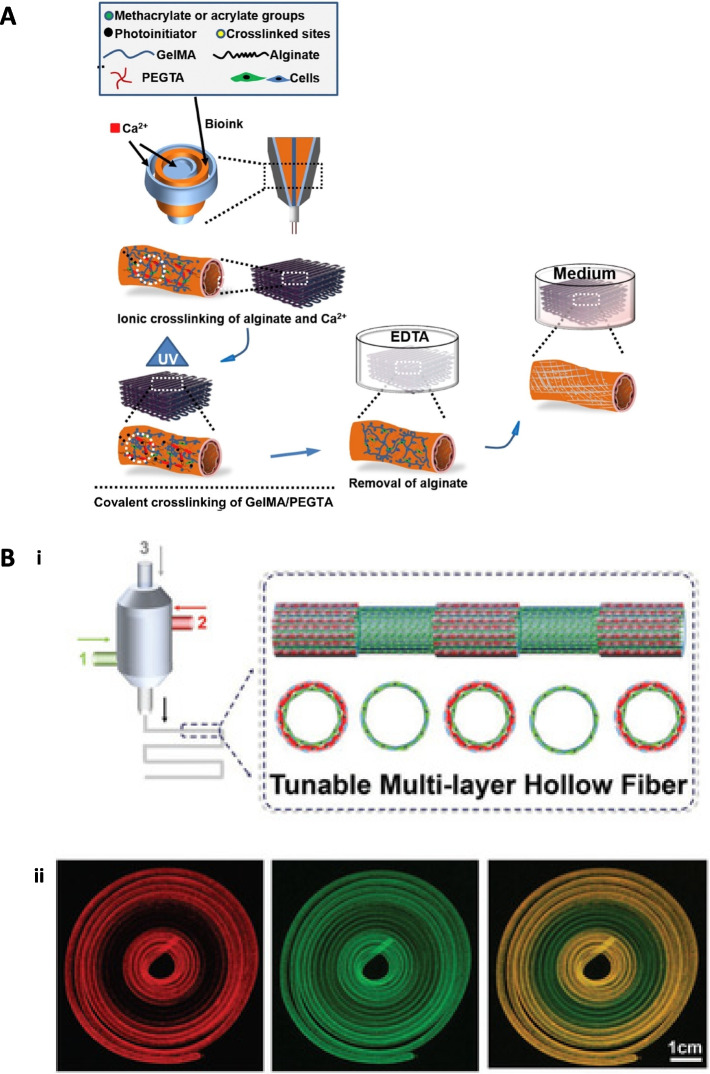
(**A**) Schematics showing the procedure of bioprinting perfusable hollow tubes with the cell-encapsulating blend bioink and subsequent vascular formation. Reproduced with permission [84]. Copyright 2016, Elsevier. (**B**) i) Schematic illustration of tuning of layers of a hollow fiber with multichannel coaxial extrusion system during a 3D bioprinting process. ii) Fluorescence confocal microscopy images showing dynamic conversion between single and double-layered hollow tubes during the bioprinting process (scale bar: 1 cm). Reproduced with permission [85]. Copyright 2018, John Wiley and Sons

3D bioprinting is an emerging technology allowing the fabrication of irregular patient-specific tubular structure vascular grafts with high precision and reproducibility. It is suitable for printing tissue-specific cells, polymers, or cells-embedded polymers, mimicking the native-like tissue makeup (Fig. 6). According to the distinct principles, 3D bioprinting techniques can be divided into inkjet-based 3D bioprinting, extrusion-based 3D bioprinting, direct laser writing, and photocuring-based 3D bioprinting is one of the earliest technologies, which uses a piezoelectric or thermal actuator to separate the biological ink with cell-embedded polymers into a series of tiny droplets, forming a layer-by-layer 3D structure [83]. Moreover, inkjet-based 3D bioprinting could be designed with more than one nozzle to print different cells simultaneously at a high speed. However, the low driving pressure of the nozzle makes it challenging to use polymers with high viscosity, resulting in relatively low mechanical properties. Low-viscosity materials such as collagen and fibrin are suitable for inkjet-based 3D bioprinting with minimal cell death [106]. Compared with the inkjet-based method, extrusion-based 3D bioprinting technology is widely used for high-viscosity materials using a pneumatically or mechanically driven nozzle [84, 107] (Fig. 6A). Instead of individual droplets, continuous fibers are extruded from the nozzle which can be switched to monolayer or multilayers, leading to better structural strength for tissue-engineered vascular grafts (Fig. 6B). It is generally formed by ionic cross-linking, temperature-induced cross-linking, and light-induced cross-linking [84, 85]. In addition, laser direct writing 3D bioprinting is considered a non-nozzle inkjet type, which also uses droplets as the basic forming unit. This technology can accurately pattern multiple cell types, such as HUVECs, in two or three dimensions [108]. The advantage of this technology is that it avoids mechanical shear damage to the cells due to non-contact between biological inkjet and the device, in contrast to the damages that occur during the inkjet-based 3D bioprinting process. However, laser direct technology is still in its early days because of the cost, manufacturing time, lack of commercial printing devices, and so on. Photocuring-based 3D bioprinting is similar to the laser direct writing method, forming a 3D structure through multiple layers of biological inks selectively cross-linked by light. The advantage of this method involves the high precision of the structure regardless of the complexity; however, UV light and its initiators cause some damage to cells.

### Cell sheet engineering

**Fig. 7:**
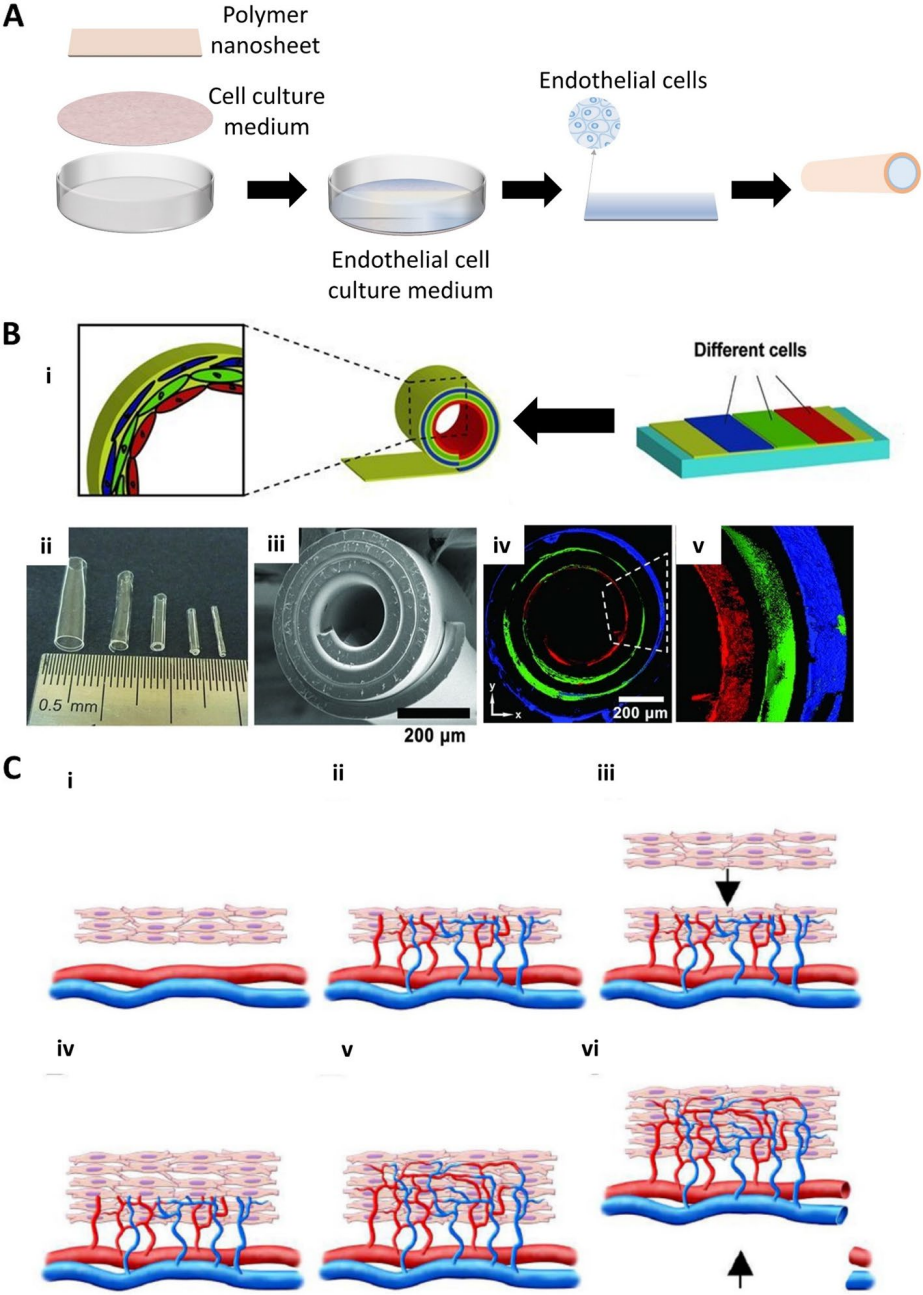
(**A**) Fundamental schematic diagrams of cell sheet engineering. (**B**) i) schematic illustrations of different cell delivery and the fabrication process of tissue-engineered vascular grafts. ii) Optical image of tubes with different diameters. The sizes of tubes are related to the thickness and stress of the membrane. iii) Scanning electron microscopy (SEM) images of a stress-induced rolling membrane with about three-layered walls. iv) Confocal images of the stress-induced rolling membrane with different cell distributions. Each color indicates a different type of cell. Red are endothelial cells (HUVECs); green: are smooth muscle cells; blue: are fibroblasts (NIH 3T3). Reproduced with permission [86]. Copyright 2012, John Wiley and Sons. (**C**) Schematic illustration of the concept used for bioengineering multilayer grafts with surgically connectable vessels. i) First graft is transplanted over a surgically accessible artery and vein. ii) The graft is supplied with new vasculature and blood directly from these existing vessels. iii, iv) After sufficient vascularization, the second graft is transplanted onto the first. v, vi) Finally, the micro-vascularized construct accompanied by available vessels harvested from the host is fully perfused by host vessels and surgically resected. Ectopic transplantation of such a graft is then possible. Reproduced with permission [111]. Copyright 2006, John Wiley and Sons

Cell sheet engineering technology develops complex and functional tissues through which the adhesion and detachment of cells can be controlled (Fig. 7A). A vascular bed with progressive stacking of three-layered cell sheets induces capillary networks containing microchannels or vascular framework within high cell density, which contributes to the anastomosis with host blood vessels (Fig. 7B) [86, 109]. Ahn et al*.* [110] developed a vascular scaffold with a smooth muscle cell sheet combined with electrospinning technology, mimicking the structural and biomechanical properties of native blood vessels. This provides high cell seeding efficiency, a more mature soft muscle layer, and good mechanical properties. If two tri-layered cell sheets are transplanted at intervals of 1 or 2 days, they will beat at the early stage followed by synchronized after one week, indicating their electrical connection (Fig. 7C) [111].

### Decellularization

**Fig. 8:**
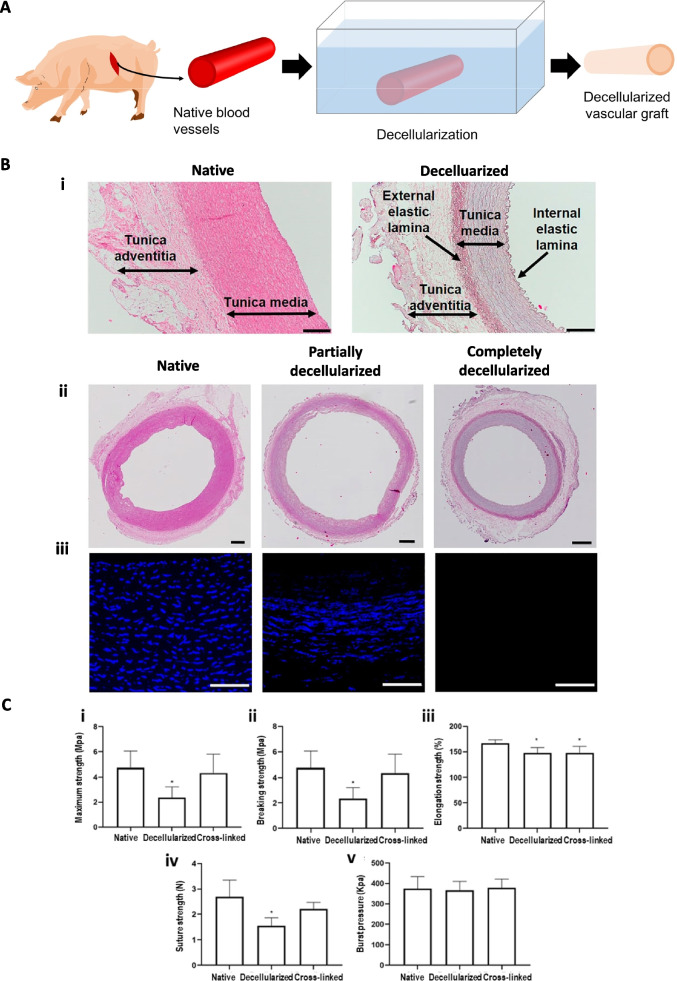
(**A**) Fundamental schematic diagrams of decellularization. (**B**) Porcine coronary artery layers and evaluation of the decellularization efficacy: i) detailed images of the native and decellularized carotid artery wall with the identification of the wall layers. In the native vessel, the internal and external elastic laminas between each layer are indistinguishable, while they can be easily identified in the decellularized scaffold. The scale bar is 200 μm. ii) Hematoxylin–eosin staining of the entire section (native, wholly and partially decellularized scaffold) – scale bar is 500 μm; iii) DAPI immunofluorescence at magnification × 40 showing a nuclear loss in the middle picture and complete cell removal on the right side) scale bar is 100 μm. Reproduced with permission [112]. Copyright 2022, Frontiers Media SA. (**C**) Biomechanical properties of the native, decellularized, and cross-linked arteries. *p < 0.05, significantly different from the native arteries. Reproduced with permission [114]. Copyright 2019, Springer Nature

Vessel decellularization reduces the immune response of a scaffold after transplantation. Some products, such as Artegraft®, ProCol®, and SynerGraft®, are available for clinical use in relatively large diameter vascular grafts (Fig. 8A) [3]. Various decellularized protocols have been extensively investigated for applications to SDVGs. Massaro et al*.* [112] proposed a new and fast decellularization protocol for porcine carotid arteries with well-preserved ECM components and good mechanical properties comparable to that of the native blood vessels, which has an excellent potential for vascular grafts transplantation (Fig. 8B). Recently, decellularized vascular grafts from human small arteries/veins were developed using detergent-enzymatic decellularization protocols. This method maintains proper functioning without thrombosis or inflammatory rejection, as well as substantial endothelialization and cell invasion after implantation [113]. In addition, cross-linking agents can be used in decellularized arteries to improve their mechanical properties and to reduce inflammatory reactions. The immobilization of heparin could effectively inhibit thrombogenesis (Fig. 8C) [114]. However, it is still not commercially available for SDVGs to treat thrombosis, infection, aneurysms, and the cost for development is higher than existing synthetic grafts.

### Coculture of cells

**Fig. 9:**
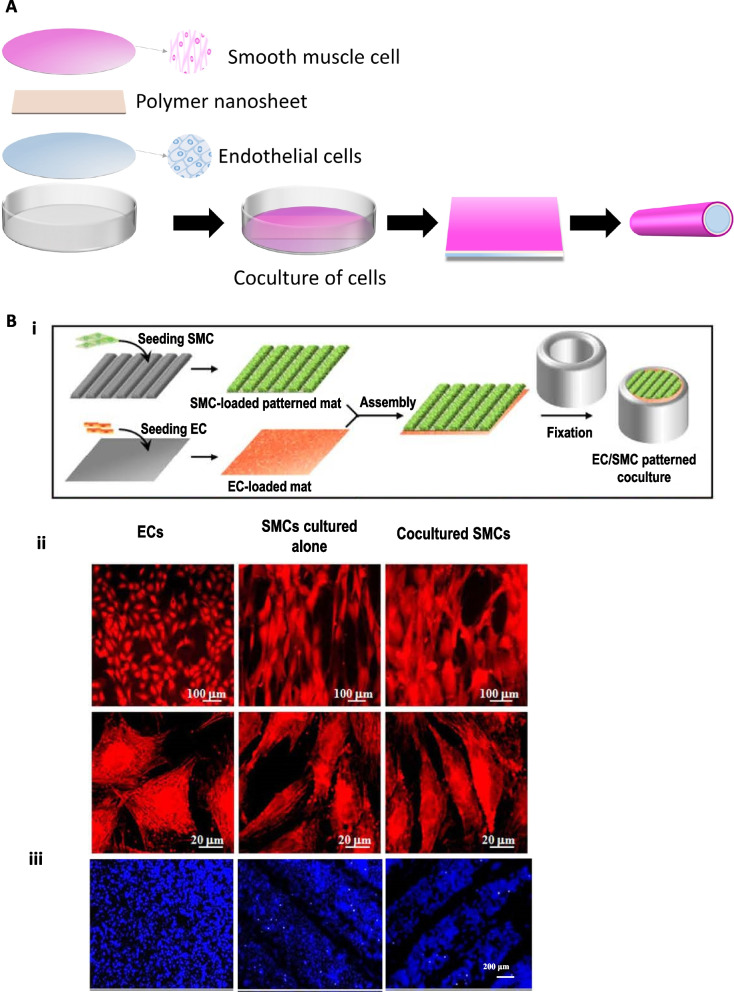
(**A**) Fundamental schematic diagrams of coculture of cells. (**B**) i) Schematic illustration of the coculture process of stacking an SMC-loaded patterned fibrous mat on an EC-loaded flat, fibrous mat, followed by fixing through two concentric glass tubes. ii) CLSM images of TRITC-phalloidin stained F-actin of ECs cocultured on fibrous mats, SMCs cultured alone, and SMCs cocultured on the patterned fibrous mat. iii) CLSM images of DAPI-stained ECs after coculture on fibrous mats, SMCs cultured alone, and SMCs cocultured on patterned fibrous mats with the ridge/groove width of 300/100 µm after incubation for seven days. Reproduced with permission [116]. Copyright 2014, John Wiley and Sons

It is crucial to establish a proper microenvironment mimicking the in vivo-like condition of blood vessels for tissue regeneration (Fig. 9A). Lu et al*.* [115] studied the effect of endothelium-releasing vasoactive factors on vascular smooth muscle cell (VSMC) proliferation in the coculture system of isolated aortic endothelial cells (AECs) and the VSMCs. They observed that the endothelin-1 concentration increased in the coculture system compared with VSMCs cultured alone. This indicates some interactions between SMCs and ECs, affecting the proliferation of SMCs but not ECs. In addition, compared with SMCs cultured alone, the coculture system with ECs on patterned fibrous scaffolds could promote the extracellular matrix production of both ECs and SMCs, providing a promising technology in the design of tissue-engineered scaffolds to construct blood vessels (Fig. 9B) [116]. Kook et al*.* [117].fabricated a 3D mesh scaffold with HUVECs and adipose-derived mesenchymal stem cells (ADSCs) along the electrospun nanofibers It is reported that HUVECs and ADSCs co-cultured PCL/gelatin nanofiber scaffolds could accelerate mature and functional micro-vessels and a luminal structure with a higher expression of vascular markers. Kuzminska et al*.* [118] obtained prostheses with EC and SMC co-culture system as well as the REDV peptide that increased the hemocompatibility and EC adhesion, but decreased platelet adhesion.

Overall, electrospinning and cell sheet engineering are advantageous for producing multilayer structure with different types of cells each layer, mimicking similar fibrous structure of native ECM to enhance cell infiltration, cell adhesion, cell proliferation, and so on, while coculture of cells offers the cellular environment between cell–cell and cell-medium interactions. Similarly, molding also provides multilayer structure of SDVGs, but needs some reinforcements to improve their mechanical properties. The merit of 3D bioprinting is to fabricate automatically customized SDVGs with complex structures in the presence of cells, however, it is challenging to apply to the mass production due to its slow processing speed. In addition, the decellularization well preserves the extracellular matrix structure as well as favorable mechanical properties of native blood vessels, however, it is difficult to perfectly decellularize which may cause immune responses.

## Conclusions and future perspective

With the development of technologies, the demand of SDVGs is also increasing for the cardiovascular reconstruction such as substitution for severely constricted or occluded blood vessels. To date, large or middles size diameter vascular grafts have achieved satisfactory results in the clinical application, and few technologies for SDVGs are already entering the stages of human clinical trials; however, it yielded limited success and it is still not commercially available for SDVGs to fulfill patient’s needs such as coronary, peripheral, and carotid arteries bypass surgery and arteriovenous access due to low patency after implantation. The key point is to resolve compliance mismatch between SDVGs and native vessels at the anastomosis site which results in intima hyperplasia, platelets adhesion, thrombus formation, stenosis, and immune system activation [119].

This review focused on the commonly used synthetic polymers used for SDVGs, and their surface modification strategies as well as various fabrication techniques for the long-term patency of SDVGs. The overall idea is to design multilayer vascular grafts, using synthetic polymers (ePTFE, PET, PU, PLA, or PCL) combined with natural polymers (collagen, elastin, gelatin, lecithin) introducing biologically active substances (peptides, vascular growth factors, antibodies, plasminogen activators) as well as specific cells (HUVECs, HUASMCs or fibroblasts) on each layer to mimic the composition of natural blood vessels as well as cellular environment in the human body [86], leading to better biocompatibility and the reduction of immune responses [23]. In terms of fabrication technologies, multilayer structures of SDVGs can be produced by electrospinning, molding, cell sheet engineering, or the combination of two or three fabrication technologies, making sure that SDVGs have adequate mechanical properties and biocompatibility. For example, electrospinning and coculture of cells could be combined to mimic ECM structure that facilitate to the infiltration, adhesion, and proliferation of specific cells. However, it is a challenge to fabricate complicated geometries via electrospinning or cell sheet engineering, whereas SDVGs with complicated geometries could be easily produced through molding or 3D bioprinting. Such an approach will combine the advantages of different biomaterials and various fabrication technologies for vascular reconstruction. In addition, decellularization of native blood vessels including allogenic and xenogeneic vessels (from porcine, bovine, etc.) is also a suitable technology for vascular reconstruction. Although it has a good ECM as well as mechanical properties, it is associated with the immune responses resulting in long-term failure.

Considering the above information of this review, it can be concluded that the production of SDVGs needs further improvement for commercialization. Future studies should optimize fabrication technologies with proper biomaterials and innovative technologies for surface modification, mimicking physiological environment in the body to the great extent. The surface modification of SDVGs should be focused on with the combination of “passive and active” strategies and their synergistic mechanisms to solve the thrombus formation in the initial stage as well as rapid reconstruction of SDVGs. For example, zwitterionic groups grafted on the synthetic polymers have anti-thrombotic effect [120], and REDV peptides grafted on SDVGs can selectively adhere endothelial cells to promote rapid endothelialization [57], which are passive strategy to prevent thrombus formation after implantation. Meanwhile, plasminogen activators, which is an active strategy, are also introduced combined with the above passive strategy to dissolve the fibrin formed at the early stage for the long-term patency of SDVGs. In addition, a better understanding of the correlation between biomaterials, surface modification, fabrication parameters, and performances would provide a mean to the create multi-functional long-term patency of SDVGs.

## Data Availability

Not applicable.

## Abbreviations

3D: Three-dimensional
SDVGs: Small-diameter vascular grafts
FGF: Fibroblast growth factor
VEGF: Vascular endothelial growth factor
HGF: Hepatocyte growth factor
ECM: Extracellular matrix
ePTFE: Expanded polytetrafluoroethylene
PET: Polyethylene terephthalate
PU: Polyurethane
PLA: Polylactic acid
PLLA: Poly-L-lactic acid
PDLA: Poly-D-lactic acid
PLGA: Poly(lactic-co-glycolic acid)
PCL: Poly(-caprolactone)
PGA: Poly (glycolic acid) (PGA)
NO: Nitric oxide
HUVECs: Human umbilical vein endothelial cells
HUASMCs: Human umbilical artery smooth muscle cells
HUAECs: Human umbilical artery endothelial cells
ADSCs: Adipose-derived mesenchymal stem cells
CHCl_3_: Chloroform
EtOH: Ethanol
DMF: N,N-dimethylformamide
THF: Tetrahydrofuran
HFIP: 1,1,1,3,3,3-Hexafluoro-2-propanol
