# Correction: Challenges and advances in materials and fabrication technologies of small-diameter vascular grafts

**DOI:** 10.1186/s40824-023-00424-4

**Published:** 2023-09-23

**Authors:** Mei-Xian Li, Qian-Qi Wei, Hui-Lin Mo, Yu Ren, Wei Zhang, Huan-Jun Lu, Yoon Ki Joung

**Affiliations:** 1 National and Local Joint Engineering Research Center of Technical Fiber Composites for Safety and Protection, Nantong University, 226019 Nantong, China; 2 School of Textile and Clothing, Nantong University, 226019 Nantong, China; 3 Center for Biomaterials, Biomedical Research Institute, Korea Institute of Science and Technology, 02792 Seoul, Republic of Korea; 4 Department of Infectious Diseases, General Hospital of Tibet Military Command, Xizang, China; 5 Institute of Special Environmental Medicine, Nantong University, 226019 Nantong, China; 6 Division of Bio-Medical Science and Technology, University of Science and Technology (UST), 217 Gajeong-ro, Yuseong-gu, 34113 Daejeon, Republic of Korea

**Biomaterials Research (2023) 27:58**

10.1186/s40824-023-00399-2

The original article [1] mistakenly affiliated several authors who have since been re-affiliated to their correct institutions. 

